# Assessment of rates of recanting and hair testing as a biological measure of drug use in a general population sample of young people

**DOI:** 10.1111/add.13645

**Published:** 2016-12-26

**Authors:** Michelle Taylor, John Sullivan, Susan M. Ring, John Macleod, Matthew Hickman

**Affiliations:** ^1^ MRC Integrative Epidemiology Unit (IEU)University of BristolBristolUK; ^2^ Social and Community MedicineUniversity of BristolBristolUK; ^3^ UK Centre for Tobacco and Alcohol Studies, School of Experimental PsychologyUniversity of BristolBristolUK; ^4^ Alere Toxicology PLcOxfordUK

**Keywords:** ALSPAC, cannabis, hair drug testing, illicit drugs, recanting, self‐report

## Abstract

**Aims:**

We investigate the extent of and factors associated with denial of previously reported cannabis and other illicit drug use, and assess the potential of hair testing for measuring substance use in general population samples.

**Design:**

Birth cohort study.

**Setting:**

United Kingdom, 1991–present.

**Participants:**

A total of 3643 participants who provided hair and self‐report measures of cannabis and other illicit drug use in the Avon Longitudinal Study of Parents and Children (ALSPAC) at age 18 years.

**Measurements:**

Denial of ever use of cannabis and other illicit drugs at age 18 following previously reported use. Positive hair drug tests for cannabis and other illicit drugs, and expected numbers of false positives and false negatives based on expected sensitivity and specificity.

**Findings:**

Cannabis and other illicit drug use was reported by 1223 and 393 individuals, respectively, before age 18 years. Of these 176 (14.4%) and 99 (25.2%), respectively, denied use at age 18. Denial of cannabis use decreased with the reporting of other substances and antisocial behaviour. Cannabis and other illicit drug use at age 18 was reported by 547 (22.5%) and 203 (8.4%) individuals, respectively. Of these, 111 (20.3%) and 13 (6.4%) were hair‐positive for cannabis and other illicit drugs, respectively. Based on hair testing for cannabis use we expect 0 [95% confidence interval (CI) = 0–169] false positives and 394 (95% CI = 323–449) false negatives compared to observed 362 potential false positives and 436 potential false negatives based on self‐report. In hair‐positive individuals, reporting the use of other substances and antisocial behaviour decreased the odds of a negative self‐report.

**Conclusions:**

Hair analysis provides an unreliable marker of substance use in general population samples. People who report more frequent substance use before age 18 are less likely to later deny previous substance use at age 18 than people who report occasional use.

## Introduction

Self‐report is the most commonly used method of collecting substance use data. However, it is prone to misclassification and could introduce bias in either direction, challenging the credibility of substance use research 1. Researchers have identified the phenomenon of recanting, or the denial of previously asserted life‐time substance use 2, 3. It has been suggested the errors encountered are less likely to be the result of intentional distortions but rather the result of poor comprehension, forgetting or even carelessness, as well as age of onset of reporting 4, 5, 6. Recanting, if not handled carefully, is likely to have a considerable impact upon our understanding of drug use and our efforts to prevent it 2. Knowledge of the extent and causes of under‐reporting and recanting could be used to adjust estimates based on survey reports 7, 8.

While self‐report measures are used widely, there is no true ‘gold standard’ for measuring illicit drug use (whereby the phenomenon of recanting is just one demonstration of the problems that can arise from using self‐report measures). Finding an alternative measure of drug use that is less prone to bias would be advantageous to epidemiological studies. Hair analysis holds potential for application to such studies as a biological measure of drug use. While urine and blood samples can be used to provide a measure of recent drug use (2–3 days for a single use of cannabis and up to 24 days for chronic use 9, 10, 11), hair analysis has a potential detection window of several months. Furthermore, hair testing is less invasive, accepted more readily in community settings 12 and can be stored at room temperature without the need for immediate processing 13. Fendrich and colleagues reported two main uses of drug testing in epidemiological studies: generating accurate prevalence measures and ‘correcting’ prevalence measures generated by self‐reported information 14. Several studies have assessed the plausibility of using hair samples as a replacement for urine in drug testing 15, 16, 17, 18, 19, sampling individuals with higher levels of consumption (e.g. prisoners 14, heroin overdose cases 15 and at‐risk users 19). These results are unlikely to be applicable to many epidemiological studies examining a general population sample.

Using data from the Avon Longitudinal Study of Parents and Children (ALSPAC), we aimed to (1) investigate the extent of recanting both cannabis and other illicit drug use; (2) assess the agreement between hair testing and self‐reported cannabis and other illicit drug use using previously reported sensitivity and specificity values to determine expected disagreement (false negative and false positives); and (3) identify factors associated with both recanting and self‐report disagreement in positive hair test results.

## Methods

### Design

To conduct these analysis, we obtained data collected from both questionnaires and clinic sessions in a large UK‐based birth cohort which was representative of the general population. Data were extracted from ALSPAC, a longitudinal study situated in Bristol, UK 20. Full details of the ALSPAC cohort are provided in the Supporting information. In brief, 14 541 pregnant women with expected delivery dates between 1 April 1991 and 31 December 1992 were recruited. These women, along with their offspring and partners, have been followed‐up ever since using both postal questionnaires and clinic sessions. This study design provides a large general population sample with measures of drug use at several time‐points.

### Study population

**Figure 1:**
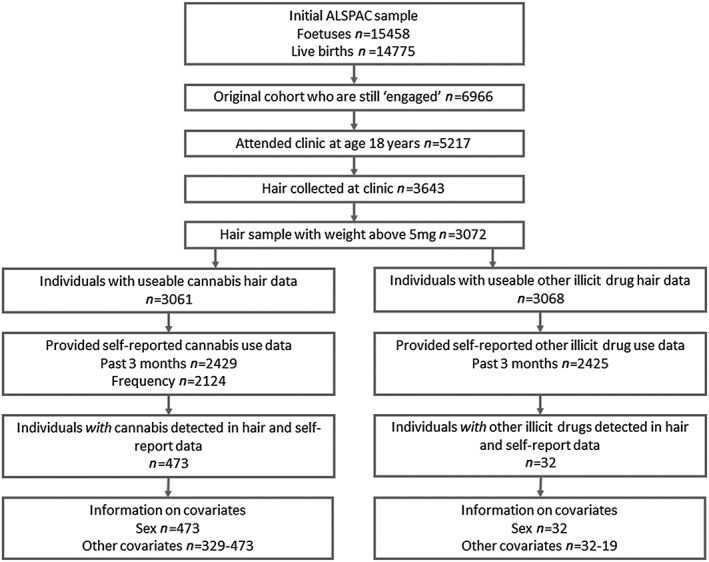
Sample derivation for analysis comparing hair drug testing and self‐report data at age 18 years

This analysis utilizes data from 5217 individuals who attended a clinic session at age 18 years (mean = 17.8 years; range = 16.3–20.0 years). A total of 3643 hair samples were collected from this clinic. Samples weighing less than 5 mg were excluded from analysis (*n* = 563). Nineteen samples did not generate useable results when testing for 11‐*nor*‐9‐carboxy‐delta‐9‐tetrahydrocannabinol **(**THC‐COOH), a metabolite of cannabis, resulting in 3061 individuals with available hair drug test data. Of these, 2429 (79.35%) completed the self‐report questions on cannabis use. Twelve samples did not generate usable results when testing for other illicit drugs and their metabolites, resulting in 3068 individuals with available hair drug test data. Of these, 2425 (79.0%) completed the self‐report questionnaires on other illicit drug use (Fig. 1). Other measures of drug use and covariates were taken from questionnaires completed at age 14 (mean = 14.3 years; range = 14.0–16.2 years) and 17 years (mean = 16.7 years; range = 16.4–18.1 years) and a clinic session at age 15 years (mean = 15.5 years, range = 14.3–17.7 years).

## Measures

### Current drug use (self‐reported)

Self‐reported measures at age 18 years included: cannabis use in the past 3 months (binary variable: yes/no); frequency of cannabis use (categorical variable: not in the past 3 months/monthly or less/two to four times per month/two to three times per week/four or more times per week); heavy cannabis use (four or more times per week) in the past 3 months (binary variable: yes/no); and other illicit drug use (cocaine, amphetamines, inhalants, sedatives, hallucinogens, opioids) in the past 3 months (binary variable: yes/no). All illicit drugs other than cannabis were combined.

### Recanting measures

In addition to the drug use measures reported above, individuals were asked about ever use of substances at age 18 years. Previously reported ever use of cannabis and other illicit drugs was also available at ages 14, 15 and 17 years (binary variables: yes/no). Recanting was defined as denying ever use of cannabis or other illicit drug at age 18 years after reporting use at any of the earlier ages (binary variables: not recanted/recanted use). Individuals who had not reported use at earlier ages were excluded from analysis.

### Hair drug measures

Measures relating to the detection of cannabis (measured using THC‐COOH) and other illicit drugs (opiates, amphetamines, cocaine, ecstasy and their metabolites) documented the results of the hair drug testing. Detection above cut‐off values ‘confirmed’ use within the past 3 months (binary variables: detected/not detected). Illicit drugs other than cannabis were grouped together. Details of the collection and extraction methods and cut‐off values for hair drug testing are provided in the Supporting information.

### Covariates/predictors

Potential covariates/predictors were based on earlier analyses 3, 19. This included a measure of past reported frequency of cannabis or other illicit drug use at ages 14, 15 and 17 years (binary variables: used fewer than five times/used five times or more times). Other drug use measures included measures of licit drugs collected at 18 years [tobacco use in the past 30 days (binary variable: yes/no); blood cotinine levels representative of smoking within the past month using a cut‐off of 10 ng/ml (these were collected at the same visit as the hair sample) 21 (binary variable: yes/no); and current alcohol use (binary variable: non‐hazardous use/hazardous or harmful use)]. Details of the extraction methods for blood cotinine levels are provided in the Supporting information. Other measures included: sex; familial social class (binary variable: I, II and III non‐manual/III manual, IV and V 22); maternal education (binary variable: CSE, vocational or O‐level/A‐level or degree); GCSE English and Mathematics results (binary variables: A*–C grade*/*D–U grade); depressive symptoms using the Short Mood and Feelings Questionnaire (SMFQ) at 18 years [dichotomized to demonstrate high (score > 11) and low (score ≤ 11) levels of depressive symptoms 23, 24]; self‐reported antisocial behaviour in the past year at 18 years [binary variable: no behaviours/one or more behaviours, where behaviours are determined using core offences in the 2005 Offending, Crime and Justice Survey (mugging, shoplifting, break and enter, selling drugs, fire‐setting, selling and buying stolen goods 25)].

### Statistical analyses

Individuals recanting previously reported cannabis and other illicit drug use were compared to individuals who did not recant use on a variety of predictors using logistic regression. Overall agreement between hair drug testing and self‐reported use of cannabis and other illicit drugs was assessed. Previously calculated sensitivity and specificity values of hair testing for THC‐COOH and other illicit drugs were used to calculate the expected number of false positives and negatives using the following equations:

$$\text{Expected false negatives}=\left(1-\text{sensitivity}\right)\times {\mathrm{SR}}_{p}$$

$$\text{Expected false positives}=\left(1-\text{specificity}\right)\times {\mathrm{SR}}_{N}$$

where *SR*_*P*_ is the number of individuals with a positive self‐report of drug use and *SR*_*N*_ is the number of individuals with a negative self‐report of drug use. This allowed for examination of the reliability of hair drug testing to this sample, as one would expect to find that potential false positives and negatives fall within the expected boundaries. Expected false positives and negatives for THC‐COOH were calculated to compare: (a) any use in the past 3 months with no use; and (b) heavy use in the past 3 months with no use or light use. The sensitivity and specificity values used for THC‐COOH (calculated by Taylor and colleagues) were: 0.28 [95% confidence interval (CI) = 0.18–0.41] and 1.00 (95% CI = 0.91–1.00), respectively, for comparing any use in the past 3 months with no use; and 0.54 (95% CI = 0.33–0.73) and 0.95 (95% CI = 0.88–0.99), respectively, for comparing heavy use with no use and light use 26. The sensitivity and specificity values used for other illicit drugs (calculated by Ledgerwood and colleagues) were 0.93 and 0.69, respectively (no confidence intervals provided) 12. Finally, within hair‐positive individuals, we tested whether those with conflicting self‐report differed on a range of covariates using logistic regression. In individuals who were hair‐positive for other illicit drugs, analyses were conducted using all available data due to the small number of individuals who tested hair‐positive. All analyses were carried out using Stata version 13 27.

## Results

### Recanting

#### Cannabis recanting

Ever use of cannabis was reported by 1223 individuals at ages 14, 15 or 17 years. Of these, 176 (14.4%) did not report ever use at age 18 and therefore recanted use. Recanting cannabis use decreased with self‐reported use of cannabis five or more times in the past [odds ratio (OR) = 0.24, 95% CI = 0.11–0.50, *P* ≤ 0.001] and with the reporting of all other substances. Self‐reported antisocial behaviour at age 18 years also reduced the odds of recanting cannabis. None of the other predictors of recanting showed evidence of an association (Table 1).

**Table 1:** Predictors of recanting use of cannabis at age 18 years using logistic regression (complete case analysis *n* = 546).

Covariates	Recanted cannabis use		Total N (%)	OR (95% CI)	P
	No (n = 505) *n* (%)	Yes (n = 41) *n* (%)			
Sex					
Male (ref)	196 (92.9)	15 (7.1)	211 (100)	1.10 (0.57–2.13)	0.778
Female	309 (92.2)	26 (7.8)	335 (100)		
Past reported cannabis use frequency					
Fewer than 5 times (ref)	230 (87.8)	32 (12.2)	262 (100)	0.24 (0.11–0.50)	≤ 0.001
5 or more times	275 (96.8)	9 (3.2)	284 (100)		
Alcohol consumption					
Non‐hazardous (ref)	182 (88.8)	23 (11.2)	205 (100)	0.44 (0.12–0.84)	0.013
Hazardous and harmful	323 (94.7)	18 (5.3)	341 (100)		
Tobacco (past 30 days)					
No (ref)	222 (86.7)	34 (13.3)	256 (100)	0.16 (0.07–0.37)	≤ 0.001
Yes	283 (97.6)	7 (2.4)	290 (100)		
Other illicit drugs (past 3 months)					
No (ref)	392 (90.7)	40 (9.3)	432 (100)	0.09 (0.01–0.64)	0.016
Yes	113 (99.1)	1 (0.9)	114 (100)		
Antisocial behaviour					
No (ref)	350 (90.7)	36 (9.3)	386 (100)	0.31 (0.12–0.81)	0.017
Yes	155 (96.9)	5 (3.1)	160 (100)		
Social class					
I, II and III non‐manual (ref)	357 (93.5)	25 (6.5)	382 (100)	1.54 (0.80–2.98)	0.195
III manual, IV and V	148 (90.2)	16 (9.8)	164 (100)		
Maternal education					
CSE, vocational, O‐level (ref)	220 (90.9)	22 (9.1)	242 (100)	0.70 (0.35–1.26)	0.213
A‐level, degree	285 (93.8)	19 (6.2)	304 (100)		
English GCSE					
D–U (ref)	60 (90.9)	6 (9.1)	66 (100)	0.79 (0.32–1.95)	0.604
A*–C	445 (92.7)	35 (7.3)	480 (100)		
Mathematics GCSE					
D–U (ref)	84 (94.4)	5 (5.6)	89 (100)	1.44 (0.55–3.77)	0.462
A*–C	421 (92.1)	36 (7.90	457 (100)		
SMFQ at 18 years					
No (ref)	372 (92.3)	31 (7.7)	403 (100)	0.90 (0.43–1.89)	0.785
Yes	133 (93.0)	10 (7.0)	143 (100)		

SMFQ (Short Moods and Feelings Questionnaire): no depressive symptoms (score ≤ 11) used as reference; GCSE = general certificate of secondary education; OR = odds ratio; CI = confidence interval.

#### Other illicit drug recanting

Ever use of other illicit drugs was reported by 393 individuals at ages 14, 15 and 17 years. Of these, 99 (25.2%) did not report ever use at age 18 and therefore recanted use. The odds of recanting other illicit drug use decreased with self‐reported use of other illicit drugs five or more times in the past (OR = 0.14, 95% CI = 0.03–0.66, *P* = 0.012) and with the reporting of other substances. There was evidence that individuals whose mother had a higher education were less likely to recant use of other illicit drugs. None of the other predictors of recanting showed evidence of an association (Supporting information, Table S1).

#### Comparison of self‐report and hair testing

Seventeen different drugs/metabolites from six drug classes were detected in at least one individual's hair (Table 2).

**Table 2:** Descriptive data of all drugs detected using hair analysis.

Drug class	Drug/metabolite	Positive cut off (ng/mg)	Total (N)	Detected (n)	Detected (%)	Range (ng/mg)
Cannabis	THC‐COOH	0.004	3065	627	20.5	0.004–0.05
Opiates	Dihydrocodeine	0.2	919	4	0.4	0.32–31.27
	Codeine	0.2	919	16	1.7	0.21–2.74
Cocaine	Cocaine	0.2	904	2	0.2	0.71–55.16
	Norcocaine	0.2	904	1	0.1	0.38
	Benzoylecgonine	0.2	904	1	0.1	0.37
Benzodiazepines	Midazolam	0.2	1043	1	0.1	0.85
	Diazepam	0.2	1043	1	0.1	0.86
	Oxazepam	0.2	1043	1	0.1	0.75
	Alprazolam	0.2	1043	2	0.2	0.38–0.38
	Desmethyldiazepam	0.2	1043	1	0.1	0.38
Methamphetamine	Methamphetamine	0.2	884	6	0.7	0.22–1.22
	MDA	0.2	884	2	0.2	0.21–0.29
	MDEA	0.2	884	2	0.2	0.45–0.46
	MBDB	0.2	884	1	0.1	0.26
	MDMA	0.2	884	14	1.6	0.24–13.89
Ketamine	Ketamine	0.2	870	7	0.8	0.28–20.70
Illicit drugs	All including cannabis	Various: see above	3068	649	21.2	NA
	All excluding cannabis	0.2	3068	32	1.3	NA

Only confirmation testing of each drug is shown, as there were no individuals who were detected in the screening phase without being detected in the confirmation stage (see hair testing methods in Supporting information). Drugs/metabolites detected in 0 individuals not shown. Opiates = morphine, A6MAM, heroin, Acetylcodeine; amphetamine; cocaine: aeme, cocaethylene; benzodiazepines: flurazepam, nitrazepam, lorazepam, temazepam, clonazepam. When excluding drugs that could be prescribed to an individual and therefore might not be used in an ‘illicit’ way, the number of individuals with other illicit drugs detected in their hair did not change, showing that these drugs were also being used alongside other illicit substances. THC‐COOH = 11‐*nor*‐9‐carboxy‐delta‐9‐tetrahydrocannabinol; MDA = 3,4‐methylenedioxyamphetamine; tenamfetamine; MDEA =3,4‐methylenedioxy‐*N*‐ethyl‐amphetamine; MBDB =1,3‐benzodioxolyl‐*N*‐methylbutanamine; MDMA = 3,4‐methylenedioxy‐methamphetamine. NA = not applicable.

#### Cannabis (THC‐COOH)

Information on cannabis use in the past 3 months was provided by 2429 individuals. Use of cannabis was reported by 547 (22.5%) individuals, 370 (67.6%) of whom reported using cannabis monthly or less, 80 (14.6%) used two to four times per month, 38 (6.9%) used two to three times per week and 55 (10.1%) used four or more times per week. Four (0.8%) did not report frequency of use. Of those reporting cannabis use in the past 3 months 111 (20.3%) had a positive hair test for cannabis, while 362 (19.2%) of those not reporting use in the past 3 months had a positive hair test (Table 3). There was minimal change to these proportions when excluding individuals with missing information on predictors used later in this analysis (complete case sample) (Supporting information, Table S2).

**Table 3:** Comparison of self‐report cannabis and other illicit drug measures with the detection of THC‐COOH (cannabis metabolite) or other illicit drugs (and metabolites) in hair.

Self‐reported Drug use	Hair analysis		Total N (%)	Expected rates of detection using hair analysis	
	Not detected n (%)	Detected n (%)		Not detected n (95% CI)	Detected n (95% CI)
Cannabis use (past 3 months)					
No	1520 (80.8)	362 (19.2)	1882 (100)	1882 (1713–1882) **394 (323–449)**	**0 (0–169)** 153 (98–224)
Yes	436 (79.7)	111 (20.3)	547 (100)		
Total	1956 (80.5)	473 (19.5)	2429 (100)		
Heavy cannabis use (past 3 months)					
No	1605 (80.8)	381 (19.2)	1986 (100)	1887 (1748–1966) **62 (36–90)**	**99 (20–238)** 73 (45–99)
Yes	104 (77.0)	31 (23.0)	135 (100)		
Total	1709 (80.6)	412 (19.4)	2121 (100)		
Frequency of cannabis use					
Never/not in the past 3 months	1276 (80.9)	302 (19.1)	1578 (100)	NA	NA
Monthly or less	302 (81.6)	68 (18.4)	370 (100)		
2–4 times per month	69 (86.3)	11 (13.7)	80 (100)		
2–3 times per week	27 (71.1)	11 (28.9)	38 (100)		
4+ times per week	35 (63.6)	20 (36.4)	55 (100)		
Total	1709 (100)	412 (100)	2121 (100)		
Use of other illicit drugs (past 3 months)					
No	2203 (99.1)	19(0.9)	2222 (100)	2066	**156**
Yes	190 (93.6)	13 (6.4)	203 (100)	63	140
Total	2393 (98.7)	32 (1.3)	2425 (100)		

Expected rates of detected calculated using previously reported sensitivity and specificity values for THC‐COOH and other illicit drugs in hair, 95% confidence intervals calculated where possible from previously reported values. Expected rates highlighted in bold type relate to the expected numbers of false positives and false negatives in the ALSPAC sample. THC‐COOH complete case sample *n* = 727; other illicit drugs using all available data. Heavy cannabis use defined as weekly cannabis use (i.e. two to three times per week or four or more times per week). THC‐COOH in hair compared to all self‐reported cannabis variables, all other illicit drugs and their compared to self‐report other illicit drug variable. CI = confidence interval; NA = not applicable.

The expected level of false positives (self‐report negative and hair‐positive) and false negatives (self‐report positive and hair‐negative) were 0 (95% CI = 0–169) and 394 (95% CI = 323–449), respectively, when comparing cannabis users with non‐users. There were 362 individuals with a positive hair test and negative self‐report (i.e. potential false positives) and 436 individuals with a negative hair test and positive self‐report (i.e. potential false negatives). Similar results were observed when comparing heavy users with light users and non‐users (Table 3). In individuals who were hair‐positive, those not reporting cannabis use were less likely to report the use of other drugs, including hazardous alcohol consumption, other illicit drugs and tobacco in the past 30 days, when compared with individuals who did report use (Table 4). They were also less likely to report antisocial behaviour at age 18 years. None of the other factors showed evidence of an association (Supporting information, Table S3).

**Table 4:** Comparison of reporting licit and illicit substance use with self‐reported cannabis use in individuals who had cannabis metabolite THC‐COOH detected in their hair using logistic regression (complete case analysis *n* = 174).

Use of other drugs	Self‐reported use (past 3 months) in those with cannabis detected in hair		Total N (%)	OR (95% CI)	P
	Yes (n = 29) n (%)	No (n = 145) n (%)			
Alcohol consumption					
Non‐hazardous	4 (4.1)	93 (95.9)	97 (100)	0.08 (0.03–0.26)	≤ 0.001
Hazardous and harmful	25 (35.5)	52 (67.5)	77 (100)		
Tobacco (past 30 days)					
No	8 (6.0)	126 (94.0)	134 (100)	0.04 (0.02–0.12)	≤ 0.001
Yes	21 (52.5)	19 (47.5)	40 (100)		
Serum cotinine (> 10 ng/ml)					
No	18 (11.4)	140 (88.6)	158 (100)	0.06 (0.02–0.20)	≤ 0.001
Yes	29 (16.7)	5 (31.30	16 (100)		
Other illicit drugs (past 3 months)					
No	18 (11.4)	140 (88.6)	158 (100)	0.07 (0.02–0.23)	≤ 0.001
Yes	11 (68.8)	5 (31.2)	16 (100)		

All measures self‐reported apart from serum cotinine levels. Serum cotinine cut‐off level of 10 ng/ml, which is representative of smoking within the past month (i.e. levels higher than that observed from passive smoking). OR = odds ratio; CI = confidence interval.

#### Other illicit drug use

Illicit drug use in the past 3 months was reported by 203 (8.4%) individuals, while 2222 (91.6%) individuals reported no use. A total of 32 of these individuals tested positive for illicit drugs and their metabolites in their hair. Of those reporting use in the past 3 months, 13 (6.4%) had a positive hair test for other illicit drugs, while 19 (0.9%) of those not reporting use had a positive hair test (Table 3). The expected levels of false positives and false negatives were 156 and 63, respectively. Nineteen individuals had a positive hair test and negative self‐report (i.e. potential false positive) and 190 individuals had a negative hair test and positive self‐report (i.e. potential false negative).

In individuals who were hair‐positive, those not reporting other illicit drug use were less likely to report the use of other drugs, including cannabis and tobacco, in the past 30 days compared to individuals who reported use (Supporting information, Table S4). These individuals were also more likely to be female and less likely to report antisocial behaviour at 18 years. None of the other factors showed evidence of an association (Supporting information, Table S5).

## Discussion

Approximately ~14 and ~25% of individuals who had previously reported use of cannabis and other illicit drugs, respectively, recanted use at age 18 years. Individuals were less likely to recant use if they reported other drug use and antisocial behaviours. This demonstrates that drug use behaviours are subject to measurement error/reporting bias, along with other behaviours that might not be deemed ‘socially acceptable’. Individuals who reported heavier use in the past were less likely to recant their use, suggesting that those who ‘experiment’ once or twice with a drug are less likely to continue reporting use later in the life‐course compared to individuals who partake in a behaviour several times. This could be influenced by both reporting and recall biases.

When comparing self‐report with hair drug‐testing information, there were more potential false positives than expected for the detection of cannabis and more potential false negatives than expected for the detection of cannabis and other illicit drugs. However, there were far fewer false positives observed than expected (19 potential versus 190 expected) for the detection of other illicit drugs. The number of potential false positives and negatives observed differ from what is expected, considering the performance of hair testing for drug use. Although there is likely to be some disagreement between self‐report and biological measures, these findings suggest that previously reported sensitivity values are overestimated for application to a general population sample.

By examining the extent of recanting in a general population sample, we have shown that self‐reported information on drug use can introduce measurement bias (and that the bias is greater for ever use compared to frequent use of illicit drugs). However, we have also demonstrated that hair drug testing is unable to provide a reliable measure of past drug use.

### Limitations of this research

This analysis has the advantage of a large sample size to examine the applicability of hair drug testing to general population samples. None the less, several limitations should be considered.

First, despite the large initial sample, the small numbers of individuals with cannabis and other illicit drugs detected in hair leaves low power for assessment of potential reasons behind any inconsistencies. This is particularly true when examining other illicit drugs, as an initial general population sample in the thousands can result in very few individuals with drugs detected in their hair. Secondly, different types and presentation forms of illicit drugs (besides combined use) show variations in time and usage pattern 28, 29, 30. Thus, combining the use of ‘other illicit drugs’ into one measure is a potential limitation. However, due to the small number of individuals both reporting the use of illicit drugs and with illicit drugs detected in their hair, it would not have been possible to examine these separately. Thirdly, while self‐reported information is unlikely to provide an accurate measure of actual exposure (e.g. strength of cannabis, how much an individual inhales, etc.), it is the most reliable measure of long‐term drug use and can be considered a ‘gold standard’ against hair drug‐testing measures. The low levels of drug use in this general population sample suggest that positive hair drug tests among people who do not self‐report cannabis use are more likely to be false positives than unreported heavy cannabis use. Here, we have demonstrated that individuals with a stronger drug history are less likely to recant self‐reported information, making it appear unlikely that individuals with heavy enough drug use to be detected are not reporting this. When examining a forensic or court sample, the opposite might apply. Finally, THC‐COOH alone was measured in hair as a marker of cannabis use. There are other metabolites of cannabis that can be detected in hair. While THC‐COOH in hair is less likely to be influenced by external contamination and might be considered the optimal metabolite, a previous analysis has shown that THC and cannabidiol show greater sensitivity and specificity 26. This analysis could have been enhanced by testing for additional cannabis metabolites.

### Comparison with literature and implications

Percy and colleagues 3 have reported several characteristics associated with recanting. The results presented here are not consistent with those reported by Percy and colleagues on gender (Percy suggested that women are less likely to recant use). However, our results are consistent when assessing other substances and antisocial behaviour. The results in our study and that of Percy and colleagues illustrate measurement error/reporting bias 31. This has wider implications for epidemiological studies on correlates of drug use, in particular studies assessing cannabis use (as this is often the only illicit drug whose use is common enough in the general population for sample effects to be estimated). Furthermore, this analysis has shown that individuals who report heavier cannabis use in the past are less likely to recant their use, therefore a measure of ever use is more likely to be misclassified and biased than frequency of use.

Reported associations using self‐reported drug use could be attributed to measurement bias influenced by perceptions of social desirability. Social desirability suggests that individuals might under‐report in order to present themselves in a more favourable way or, conversely, over‐report to ‘show off’. This over‐ or under‐reporting would be dependent upon that individual's acceptability of the behaviour 32. This theory is supported by our results showing that the use of several drugs and recanting of cannabis and other illicit drugs are associated negatively. Previous literature suggests that under‐reporting varies by drug 33, with cannabis users being less likely to under‐report. Our results are in agreement with this, with a higher percentage of ever illicit drug users recanting use.

Two studies have assessed previously the use of hair analysis as a biological measure of drug use in a general population sample. Frendrich and colleagues 14 reported higher rates of cannabis use from self‐report than from hair testing. However, we have reported higher prevalence rates from self‐reported other illicit drug use. Ledgerwood and colleagues 12 reported that self‐report and hair testing generally met good, but not excellent, agreement. These previous studies had small samples sizes in their analysis (583 and 613, respectively) in comparison to the 2452 individuals used in this study. The results reported here, along with results published elsewhere, suggest that using hair as a biomarker of drug use for alcohol 34, cannabis and other illicit drugs provides a less reliable measure than self‐report data.

## Conclusion

We have demonstrated the problem of recanting substance use in adolescents in the general population and shown that individuals recanting reporting are less likely to report the use of other substances, antisocial behaviour and depressive symptoms. However, despite the known problems with self‐reported data, our findings suggest that hair is an unreliable measure of substance use in a general population due to many factors, among them purity of drug consumed and differences in individuals’ metabolism. It is therefore not a viable tool in many epidemiological studies such as cohorts.

## Declaration of interests

None.

## Supporting information


**Table S1** Predictors of recanting use of other illicit drugs at age 18 years using logistic regression
**Table S2** Comparison of self‐report cannabis and other illicit drug measures with the detection of THC‐COOH (cannabis metabolite) or other illicit drugs (and their metabolites) in hair (complete case sample)
**Table S3** Comparison of socio‐economic and behavioural measures and reporting cannabis use (past 3 months) in individuals who had THC‐COOH (cannabis metabolite) detected in their hair using logistic regression
**Table S4** Comparison of reporting licit and illicit substance use with self‐reported other illicit drug use in individuals who had other illicit drugs (and metabolites) detected in their hair (results of logistic regression) using all available data
**Table S5** Comparison of socio‐economic and behavioural measures and reporting other illicit drug use (past 3 months) in individuals who had other illicit drugs (and metabolites) detected in their hair (results from logistic regression) using all available data

Data 1. Supporting info item
