# Supplementary material for: Assessment of rates of recanting and hair testing as a biological measure of drug use in a general population sample of young people
Source: Addiction. 2016 Dec 26;112(3):477–85. doi: 10.1111/add.13645 (PMC5324538; doi:10.1111/add.13645)
Supplement: Supplementary file 1 — Data 1. Supporting info item [file ADD-112-477-s001.docx]

**Supplementary Methods**

**Avon Longitudinal Study of Parents and Children (ALSPAC)**

The Avon Longitudinal Study of Parents and Children (ALSPAC) is a UK population-based study which aims to investigate environmental and genetic influences on the health and development of children (1, 2). Pregnant women residing in the former Avon Health Authority in south-west England who had an estimated date of delivery between 1 April 1991 and 31 December 1992 were invited to take part, resulting in a cohort of 14,541 pregnancies and 13,978 children alive at 12 months of age (excluding triplets and quads). Ethical approval for this study was obtained from the ALSPAC Law and Ethics Committee and the Local Research Ethics Committees. The representative nature of the ALSPAC sample has been investigated by comparison with the 1991 National Census data of mothers with infants under 1 year of age who were residents in the county of Avon. The ALSPAC sample had a slightly greater proportion of mothers who were married or cohabiting, who were owner-occupiers and who had a car in the household. The study had a smaller proportion of ethnic minority mothers. The ALSPAC study website contains details of all the data that are available through a fully searchable data dictionary ([www.bris.ac.uk/alspac/researchers/data-access/data-dictionary/](http://www.bris.ac.uk/alspac/researchers/data-access/data-dictionary/)).

### Collection and storage of hair

Participants were asked questions about whether they have dyed their hair, used products or straighteners. Participants were asked to dip their head forward to make it easier to find a sample just about the hairline. A few strands (ideally about 2mm across and at least 3cm long) were selected and twisted to ensure the sample started at the root. Hair was cut neatly at the scalp and the bundle of hair was placed into a foil strip. The foil strip was folded and sealed, and then placed in an envelope that was also sealed. The sample was stored at room temperature until analysed.

### Extraction method for hair drug measures

Hair samples were analysed by Alere Toxicology, Cardiff, UK.

*Sample preparation:* Two 1ml methanol washes were performed on the hair with each wash being removed to waste. An additional aliquot of methanol was added and the tubes placed in an unheated ultrasonic bath overnight in 1ml acidified methanol. Following ultrasonication, the hair sample extract was then placed in a clean test tube. The methanol was then removed and samples dried down. The dried extract was reconstituted in phosphate buffer and submitted to solid phase extraction prior to instrumental analysis.

*Solid Phase Extraction (SPE)*: SPE was used to prepare sample for analysis using a Waters 1ml HCX column. The column was primed with 1ml methanol and conditioned with phosphate buffer. 0.5ml of sample was added to each SPE tube and eluted at a flow not exceeding 1ml/minute. Cannabinoids were eluted with hexane and ethyl acetate (80:20 v/v) under gravity and collected into autosampler vials.

*Instrumentation and analysis:* Samples were screened using homogenous Enzyme Immunoassays (HEIA) after overnight sonication followed by a chemical digest and liquid/liquid extraction. Immunoassays for opiates, cocaine, amphetamine, methamphetamine, cannabis, benzodiazepines, methadone, and ketamine were run on al Ilab clinical chemistry analyser. The cut-off concentration values were 0.5ng/mg for all analytes except cannabis with a cut off of 0.1ng/mg.

Hair samples above 10mg in weight were analysed in the quantitative confirmation stage.

Cannabis was confirmed on a Agilent 7890 GC with an Agilent LTM series II system coupled to an agent 7000 QQQ (inlet temperature = 280°C; detector temperature = 180°C; injection volume = 1μL; split/splitless; flow rate = 1ml/min). The column used was a HP-5MS column (15M x 0.25 x 0.25μm) and nitial column temperature was set to 70°C for 1 minute and increased to 300°C. Cut-off concentration for cannabis (11-nor-9-carboxy-delta-9-tetrahydrocannabinol (THC-COOH)) was 0.4pg/mg (retention time = 5.51 min; transition = 371.2 to 305.2; Quan ions = 305; detector voltage = 2000v; collision energy = 12v).

Opiates, cocaine, amphetamine, methamphetamine, benzodiazepines, methadone and ketamine were confirmed on a Water Aquity Ultra Performance LC with an Aquity UPLC C18 column (1.7μm, 1.2 x 100mm column) coupled to a Waters TQ Detector (injection volume = 10μL; flow rate = 0.5ml/min; mobile phase A = 0.1% Formic Acid in Water. Mobile phase A was 0.1% Formic Acid in water and Mobile Phase B was 0.1% Formic Acid in Acetonitrile:

| Time (Min) | Flow (ml/min) | MP %A | MP %B |
| --- | --- | --- | --- |
| Initial | 0.4 | 100 | 0 |
| 0.5 | 0.4 | 99 | 1 |
| 1.5 | 0.4 | 90 | 10 |
| 3.5 | 0.4 | 80 | 20 |
| 5 | 0.4 | 60 | 40 |
| 6.5 | 0.4 | 20 | 80 |
| 7 | 0.4 | 20 | 80 |
| 7.7 | 0.4 | 100 | 0 |
| 8 | 0.4 | 100 | 0 |

Cut-off concentrations for LC-MS analysis was 0.2ng/mg.

*MS analyser parameters of LC:*

Source (ES+) settings: Capillary (kV) 0.50; Cone (V) 40.00; Extractor (V) 2.00 RF (V) 0.30; Source Temperature (°C) 150; Desolvation Temperature (°C) 450; Cone Gas Flow (L/Hr) 20; Desolvation Gas Flow (L/Hr) 80; Collision Gas Flow (mL/Min) 0.25 )

Analyser settings: LM 1 Resolution 13.00; HM 1 Resolution 13.00; Ion Energy 1 0.50; MS Mode Entrance 50.00; MS Mode Collision Energy 3.00; MS Mode Exit 50.00; MSMS Mode Entrance 1.00; MSMS Mode Collision Energy 15.00; MSMS Mode Exit 0.50; LM 2 Resolution 13.00; HM 2 Resolution 13.00; Ion Energy 2 1.00; Gain 1.20; Multiplier 0.00; Autotune Reservoir B; Active Reservoir A

Pressure gauges: Collision Cell Pressure (mbar) 9.785722e-005

*Compound specific MS parameters for LC:*

| Compound Name | Quantification Trace | Predicted RT | Response Type | Response Uses | Polynomial Type | Calibration Origin |
| --- | --- | --- | --- | --- | --- | --- |
| AEME | 182.1>122.1 | 1.88 | Relative IS | Area | Linear | Include |
| Morphine | 286.1 > 153.1 | 1.77 | Relative IS | Area | Linear | Include |
| Codeine | 300.1>165.1 | 2.52 | Relative IS | Area | Linear | Include |
| Dihydrocodeine | 302.1>245.1 | 2.46 | Relative IS | Area | Linear | Include |
| Amphetamine | 136.1 > 119.1 | 2.74 | Relative IS | Area | Linear | Include |
| Methamphetamine | 150.1 > 91.1 | 2.95 | Relative IS | Area | Linear | Include |
| MDA | 180.1 > 163.1 | 2.84 | Relative IS | Area | Linear | Include |
| MDMA | 194.1 > 163.1 | 3.01 | Relative IS | Area | Linear | Include |
| 6MAM | 328.1>165.1 | 2.69 | Relative IS | Area | Linear | Include |
| Mephedrone | 178.1 > 160.1 | 3.045 | Relative IS | Area | Linear | Include |
| MDEA | 208.1 > 163.1 | 3.34 | Relative IS | Area | Linear | Include |
| MBDB | 208.1 > 177.1 | 3.63 | Relative IS | Area | Linear | Include |
| Benzoylecgonine | 290.1>168.1 | 3.55 | Relative IS | Area | Linear | Include |
| Ketamine | 238.1 > 220.1 | 3.185 | Relative IS | Area | Linear | Include |
| Cocaine | 304.1>182.1 | 4.465 | Relative IS | Area | Linear | Include |
| Norcocaine | 290.1>168.1 | 4.6 | Relative IS | Area | Linear | Include |
| 6-Acetylcodeine | 342.1 > 225.1 | 4.02 | Relative IS | Area | Linear | Include |
| Cocaethylene | 318.1>196.1 | 4.935 | Relative IS | Area | Linear | Include |
| Chlordiazepoxide | 300.1 > 283.1 | 4.81 | Relative IS | Area | Linear | Include |
| Midazolam | 326.1>291.1 | 5.23 | Relative IS | Area | Linear | Include |
| Flurazepam | 388.1>315.1 | 5.32 | Relative IS | Area | Linear | Include |
| EDDP | 278.1>234.1 | 5.53 | Relative IS | Area | Linear | Include |
| Methadone | 310.1>265.1 | 5.84 | Relative IS | Area | Linear | Include |
| Nordiazepam | 271.1>140.1 | 6.06 | Relative IS | Area | Linear | Include |
| Oxazepam | 287.1 > 268.9 | 5.95 | Relative IS | Area | Linear | Include |
| Nitrazepam | 282.1>236.1 | 5.81 | Relative IS | Area | Linear | Include |
| Clonazepam | 316.1>270.1 | 5.99 | Relative IS | Area | Linear | Include |
| Diazepam | 285.1>154.1 | 6.43 | Relative IS | Area | Linear | Include |
| Phenazepam | 351.1>186.1 | 6.25 | Relative IS | Area | Linear | Include |
| Temazepam | 301>255 | 6.23 | Relative IS | Area | Linear | Include |
| Nordiazepam-d5 | 276.1>140.1 | 6.05 | Relative IS | Area | Linear | Include |
| Oxazepam-d5 | 292>246 | 5.91 | Relative IS | Area | Linear | Include |
| Temazepam-d5 | 306>260 | 6.23 | Relative IS | Area | Linear | Include |
| Diazepam-d5 | 290.1>198.1 | 6.44 | Relative IS | Area | Linear | Include |
| Chlorodiazepoxide-d5 | 305.1 > 232.1 | 4.79 | Relative IS | Area | Linear | Include |
| AEME-d3 | 185.1 > 125.1 | 1.88 | Relative IS | Area | Linear | Include |
| Cocaine-d3 | 307.1 > 185.1 | 4.465 | Relative IS | Area | Linear | Include |
| MDMA-d5 | 199.1 > 165.1 | 3.01 | Relative IS | Area | Linear | Include |
| Methamphetamine-d9 | 159.1 > 125.1 | 2.93 | Relative IS | Area | Linear | Include |
| Amphetamine-d10 | 146.1 > 129.1 | 2.71 | Relative IS | Area | Linear | Include |
| MDA-d5 | 185.1 > 168.1 | 2.82 | Relative IS | Area | Linear | Include |
| Ketamine-D4 | 242.1 > 224.1 | 3.195 | Relative IS | Area | Linear | Include |
| EDDP-d3 | 281.1>234.1 | 5.52 | Relative IS | Area | Linear | Include |
| Methadone-d9 | 319.1>105.1 | 5.85 | Relative IS | Area | Linear | Include |
| 6MAM-d6 | 334.1 > 165.1 | 2.69 | Relative IS | Area | Linear | Include |
| Morphine-d3 | 289.1 > 153.1 | 1.76 | Relative IS | Area | Linear | Include |
| Dihydrocodeine-d6 | 308.1>202.1 | 2.44 | Relative IS | Area | Linear | Include |
| Benzoylecgonine-d3 | 293.1 > 171.1 | 3.55 | Relative IS | Area | Linear | Include |
| Cocaethylene-d3 | 321.1>199.1 | 4.63 | Relative IS | Area | Linear | Include |

### Extraction method for blood (plasma cotinine measures

Cotinine was assayed from ethylenediaminetetraacetic acid plasma samples taken in a clinical assessment at age 18 years. Plasma samples were stored at -80 °C and allowed to thaw at room temperature before use. Cotinine was measured using the Cozart Cotinine Enzyme Immunoassay (Alere Toxicology UK, Abingdon) serum kit (M155B1). All samples, calibrators, and controls were brought to room temperature before use and were run in duplicate. Where required, samples were diluted using cotinine-free serum (fetal calf serum). Absorbance was measured spectrophotometrically at a wavelength of 450nm. The lowest calibrator used was 0.5ng/ml serum, and values below this were treated as undetectable/null (0ng/ml serum). Cotinine concentration are expressed as nanogram per millilitre of serum.

**References:**

1. Boyd A., Golding J., Macleod J., Lawlor D. A., Fraser A., Henderson J. et al. Cohort Profile: The 'Children of the 90s'--the index offspring of the Avon Longitudinal Study of Parents and Children, Int J Epidemiol 2012.

2. Golding J., Pembrey M., Jones R., Team A. S. ALSPAC--the Avon Longitudinal Study of Parents and Children. I. Study methodology, Paediatr Perinat Epidemiol 2001: 15: 74-87.

**Table S1: Predictors of recanting use of other illicit drugs at age 18 years using logistic regression**

| **Covariates** | **Complete Case Analysis (N=121)** | | | | |
| --- | --- | --- | --- | --- | --- |
|  | **Recanted illicit drug use** | | **Total**  **N (%)** | **OR**  **(95% CI)** | **P** |
|  | **No**  **(N = 102)**  **N (%)** | **Yes**  **(N = 19)**  **N (%)** |  |  |  |
| ***Sex***  Male *(ref)*  Female | 40 (85.1)  62 (83.8) | 7 (14.9)  12 (16.2) | 47 (100)  74 (100) | 1.11  (0.40-3.06) | 0.846 |
| ***Past reported other illicit drug use frequency***  Less than 5 times *(ref)*  5 or more times | 56 (76.7)  46 (95.8) | 17 (23.3)  2 (4.2) | 73 (100)  48 (100) | 0.14  (0.03-0.66) | 0.012 |
| ***Alcohol consumption***  Non-hazardous *(ref)*  Hazardous and harmful | 27 (73.0)  75 (89.3) | 10 (27.0)  9 (10.7) | 37 (100)  84 (100) | 0.32  (0.12-0.89) | 0.028 |
| ***Tobacco (past 30 days)***  No *(ref)*  Yes | 27 (79.4)  75 (86.2) | 7 (20.6)  12 (13.8) | 34 (100)  87 (100) | 0.62  (0.23-1.74) | 0.361 |
| ***Cannabis (past 3 months)***  No *(ref)*  Yes | 29 (70.7)  73 (91.2) | 12 (29.3)  7 (8.8) | 41 (100)  80 (100) | 0.23  (0.08-0.65) | 0.005 |
| ***Antisocial behaviour***  No *(ref)*  Yes | 54 (80.6)  48 (88.9) | 13 (19.4)  6 (11.1) | 67 (100)  54 (100) | 0.52  (0.18-1.48) | 0.220 |
| ***Social Class***  I, II and III non-manual *(ref)*  III manual, IV and V | 70 (85.4)  32 (82.1) | 12 (14.60  7 (17.9) | 82 (100)  39 (100) | 1.28  (0.46-3.56) | 0.641 |
| ***Maternal Education***  CSE, Vocational, O level *(ref)*  A level, degree | 41 (75.9)  61 (91.0) | 13 (24.1)  6 (9.0) | 54 (100)  67 (100) | 0.31  (0.11-0.89) | 0.029 |
| ***English GCSE***  D – U *(ref)*  A* - C | 17 (85.0)  85 (84.20 | 3 (15.0)  16 (15.8) | 20 (100)  101 (100) | 1.06  (0.28-4.09) | 0.925 |
| ***Mathematics GCSE***  D – U *(ref)*  A* - C | 21 (84.0)  81 (84.4) | 4 (16.0)  15 (15.6) | 25 (100)  96 (100) | 0.97  (0.29-3.25) | 0.964 |
| ***SMFQ at 18 years***  No *(ref)*  Yes | 69 (84.2)  33 (84.6) | 13 (15.9)  6 (15.4) | 82 (100)  39 (100) | 0.97  (0.34-2.77) | 0.947 |

SMFQ (Short Moods and Feeling Questionnaire): no depressive symptoms (score ≤11) used as reference category

**Table S2: Comparison of self-report cannabis and other illicit drug measures with the detection of THC-COOH (cannabis metabolite) or other illicit drugs (and their metabolites) in hair (complete case sample)**

| **Self-reported Drug Use** | **Hair Analysis** | | **Total**  **N (%)** |
| --- | --- | --- | --- |
|  | **Not Detected**  **N (%)** | **Detected**  **N (%)** |  |
| ***Cannabis use (past 3 months)***  No  Yes  Total | 454 (79.2)  125 (81.2)  579 (79.6) | 119 (20.8)  29 (18.8)  148 (20.4) | 573 (100)  154 (100)  727 (100) |
| ***Heavy cannabis use (past 3 months)***  No  Yes  Total | 552 (79.4)  27 (84.4)  579 (79.6) | 143 (20.6)  5 (15.6)  148 (20.4) | 695 (100)  32 (100)  727 (100) |
| ***Frequency of cannabis use***  Never/not in the past 3 months  Monthly or less  2-4 times per month  2-3 times per week  4+ times per week  Total | 454 (79.2)  93 (80.9)  22 (91.7)  5 (71.4)  5 (62.5)  579 (79.6) | 119 (20.8)  22 (19.1)  2 (8.3)  2 (28.6)  3 (37.5)  148 (20.4) | 573 (100)  115 (100)  24 (100)  7 (100)  8 (100)  727 (100) |

**Table S3: Comparison of socio-economic and behavioural measures and reporting cannabis use (past 3 months) in individuals who had THC-COOH (cannabis metabolite) detected in their hair using logistic regression**

| **Self-reported use of cannabis** | **Self-reported ever use in those with cannabis detected in hair** | | **Total** | **OR**  **(95% CI)** | **P**  **value** |
| --- | --- | --- | --- | --- | --- |
|  | **Yes** | **No** |  |  |  |
| **Sex**  *Male*  *Female*  *Total* | 12 (20.7)  17 (14.7) | 46 (79.3)  99 (85.3) | 58 (100)  116 (100) | 1.52  (0.67-3.44) | 0.316 |
| **Antisocial behaviour**  *No*  *Yes*  *Total* | 19 (12.5)  10 (45.5) | 133 (87.5)  12 (54.5) | 152 (100)  22 (100) | 0.17  (0.07-0.45) | ≤0.001 |
| **Social Class**  *I, II and III non-manual*  *III manual, IV and V*  *Total* | 22 (19.8)  7 (11.1) | 89 (80.2)  56 (88.9) | 111 (100)  63 (100) | 1.98  (0.79-4.93) | 0.144 |
| **Maternal Education**  *CSE, Vocational, O level*  *A level, degree*  *Total* | 14 (16.5)  15 (16.9) | 71 (83.5)  74 (83.1) | 85 (100)  89 (100) | 0.97  (0.44-2.16) | 0.946 |
| **English GCSE**  *D – U (ref)*  *A* - C*  *Total* | 5 (20.8)  24 (16.0) | 19 (79.2)  126 (84.0) | 24 (100)  150 (100) | 1.49  (0.81-2.74) | 0.203 |
| **Mathematics GCSE**  *D – U (ref)*  *A* - C*  *Total* | 4 (22.2)  25 (16.0) | 14 (77.8)  131 (84.0) | 18 (100)  156 (100) | 1.38  (0.47-4.09) | 0.557 |
| **SMFQ at 18 years**  *No*  *Yes*  *Total* | 22 (15.2)  7 (24.1) | 123 (84.8)  22 (75.9) | 145 (100)  29 (100) | 1.50  (0.46-4.92) | 0.507 |

**Table S4: Comparison of reporting licit and illicit substance use with self-reported other illicit drug use in individuals who had other illicit drugs (and metabolites) detected in their hair (results of logistic regression) using all available data**

| **Use of other drugs** | **Self-reported use (past 3 months) in those with other illicit drugs detected in hair** | | **Total**  **N (%)** | **OR**  **(95% CI)** | **P Value** |
| --- | --- | --- | --- | --- | --- |
|  | **Yes**  **N (%)** | **No**  **N (%)** |  |  |  |
| ***Alcohol consumption***  Non-hazardous  Hazardous and harmful  Total | 4 (25.0)  7 (50.0)  11 (36.7) | 12 (75.0)  7 (50.0)  19 (63.3) | 16 (100)  14 (100)  30 (100) | 0.33  (0.07-1.59) | 0.155 |
| ***Tobacco (past 30 days)***  No  Yes  Total | 1 (6.7)  12 (70.6)  13 (40.6) | 14 (93.3)  5 (29.4)  19 (59.4) | 15 (100)  17 (100)  32 (100) | 0.03  (<0.01-0.29) | 0.001 |
| ***Serum cotinine (>10ng/ml)***  No  Yes  Total | 2 (14.3)  4 (80.0)  6 (31.6) | 12 (85.7)  1 (20.0)  13 (68.4) | 14 (100)  5 (100)  19 (100) | 0.04  (0.01-0.59) | 0.007 |
| ***Cannabis (past 3 months)***  No  Yes  Total | 1 (6.7)  12 (70.6)  13 (40.6) | 14 (93.3)  5 (29.4)  19 (59.4) | 15 (100)  17 (100)  32 (100) | 0.03  (0.00-0.29) | 0.001 |

All measures self-reported apart from serum cotinine levels

Serum cotinine cut off level of 10ng/ml which is representative of smoking within the past month (i.e. levels higher than that observed from passive smoking)

**Table S5: Comparison of socio-economic and behavioural measures and reporting other illicit drug use (past 3 months) in individuals who had other illicit drugs (and metabolites) detected in their hair (results from logistic regression) using all available data**

| **Self-reported use of cannabis** | **Self-reported ever use in those with cannabis detected in hair** | | **Total** | **OR**  **(95% CI)** | **p** |
| --- | --- | --- | --- | --- | --- |
|  | **Yes** | **No** |  |  |  |
| ***Sex***  Male  Female  Total | 9 (60.0)  4 (23.5)  13 (40.6) | 6 (40.0)  13 (76.5)  19 (59.4) | 15 (100)  17 (100)  32 (100) | 4.88  (1.06-22.38) | 0.034 |
| ***Antisocial behaviour***  No  Yes  Total | 3 (16.7)  10 (71.4)  13 (40.6) | 15 (83.3)  4 (28.6)  19 (59.4) | 18 (100)  14 (100)  32 (100) | 0.08  (0.01-0.44) | 0.004 |
| ***Social Class***  I, II and III non-manual  III manual, IV and V  Total | 7 (41.2)  3 (30.0)  10 (37.0) | 10 (58.8)  7 (70.0)  17 (63.0) | 17 (100)  10 (100)  27 (100) | 1.63  (0.31-8.61) | 0.559 |
| ***Maternal Education***  CSE, Vocational, O level  A level, degree  Total | 6 (33.3)  4 (44.4)  10 (37.0) | 12 (66.7)  5 (55.6)  17 (63.0) | 18 (100)  9 (100)  27 (100) | 0.63  (0.12-3.22) | 0.575 |
| ***English GCSE***  D – U *(ref)*  A* - C  Total | 9 (60.0)  7 (35.0)  10 (40.0) | 2 (40.0)  13 (65.0)  16 (60.0) | 5 (100)  20 (100)  25 (100) | 2.79  (0.37-20.82) | 0.312 |
| ***Mathematics GCSE***  D – U *(ref)*  A* - C  Total | 1 (33.3)  9 (37.5)  10 (37.0) | 2 (66.7)  15 (62.5)  17 (63.0) | 3 (100)  24 (100)  27 (100) | 0.83  (0.07-10.55) | 0.887 |
| ***SMFQ at 18 years***  No  Yes  Total | 4 (25.0)  4 (50.0)  8 (33.3) | 12 (75.0)  4 (50.0)  16 (66.7) | 16 (100)  8 (100)  24 (100) | 0.33  (0.06-1.99) | 0.226 |

SMFQ (Short Moods and Feeling Questionnaire): no depressive symptoms (score ≤11) used as reference category
